# Supplementary material for: Causal interplay between lactose intolerance and gut microbiota: a combined bidirectional Mendelian randomization and in vivo validation study
Source: Front Nutr. 2026 Jun 1;13:1803337. doi: 10.3389/fnut.2026.1803337 (PMC13265576; doi:10.3389/fnut.2026.1803337)
Supplement: Supplementary file 1 [file Data_Sheet_1.zip › supplementary materials/Forward/leave one out cross validation/ebi-a-GCST90027540.finngen_R12_E4_LACTONAS.pdf]

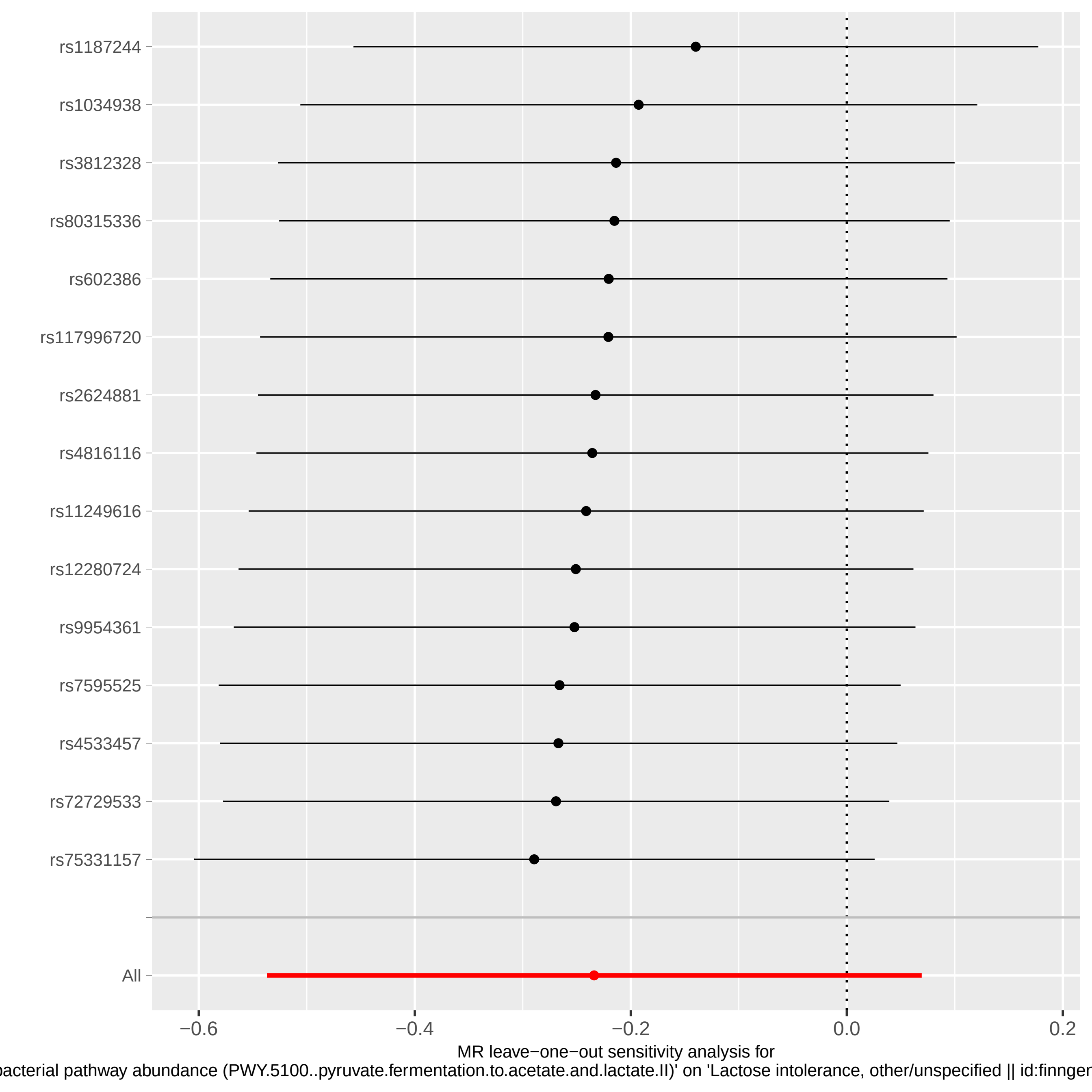

rs1187244
rs1034938
rs3812328
rs80315336
rs602386
rs117996720
rs2624881
rs4816116
rs11249616
rs12280724
rs9954361
rs7595525
rs4533457
rs72729533
rs75331157
All
−0.6
−0.4
−0.2
0.0
0.2
MR leave−one−out sensitivity analysis for
acterial pathway abundance (PWY.5100..pyruvate.fermentation.to.acetate.and.lactate.II)' on 'Lactose intolerance, other/unspecified || id:finnger
